# Climate-Driven Crop Yield and Yield Variability and Climate Change Impacts on the U.S. Great Plains Agricultural Production

**DOI:** 10.1038/s41598-018-21848-2

**Published:** 2018-02-22

**Authors:** Meetpal S. Kukal, Suat Irmak

**Affiliations:** University of Nebraska-Lincoln, Lincoln, NE 68583 USA

## Abstract

Climate variability and trends affect global crop yields and are characterized as highly dependent on location, crop type, and irrigation. U.S. Great Plains, due to its significance in national food production, evident climate variability, and extensive irrigation is an ideal region of investigation for climate impacts on food production. This paper evaluates climate impacts on maize, sorghum, and soybean yields and effect of irrigation for individual counties in this region by employing extensive crop yield and climate datasets from 1968–2013. Variability in crop yields was a quarter of the regional average yields, with a quarter of this variability explained by climate variability, and temperature and precipitation explained these in singularity or combination at different locations. Observed temperature trend was beneficial for maize yields, but detrimental for sorghum and soybean yields, whereas observed precipitation trend was beneficial for all three crops. Irrigated yields demonstrated increased robustness and an effective mitigation strategy against climate impacts than their non-irrigated counterparts by a considerable fraction. The information, data, and maps provided can serve as an assessment guide for planners, managers, and policy- and decision makers to prioritize agricultural resilience efforts and resource allocation or re-allocation in the regions that exhibit risk from climate variability.

## Introduction

**Figure 1:**
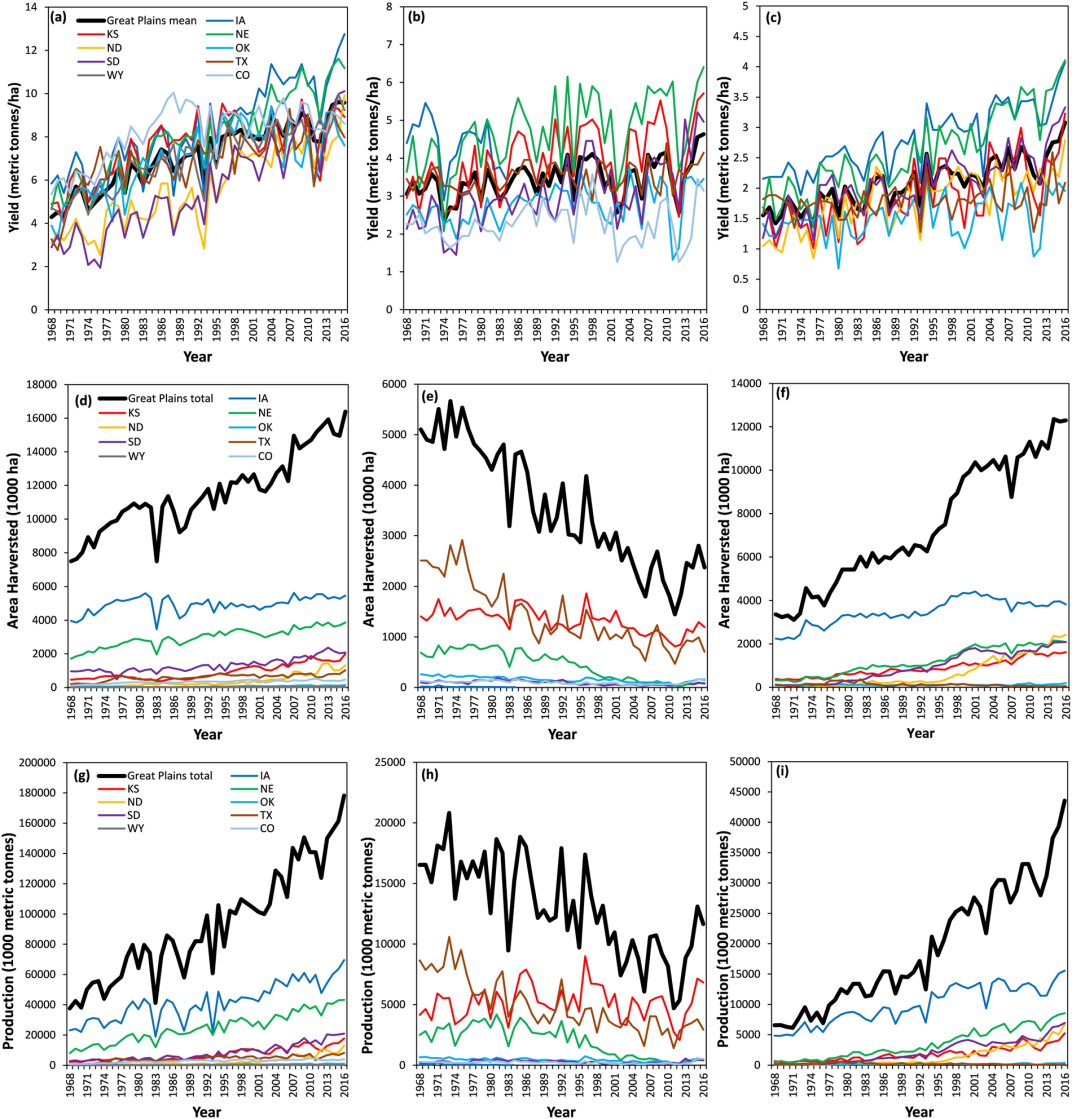
State-specific trends in (**a**) Maize yield (**b**) Sorghum yield (**c**) Soybean yield (**d**) Maize harvested area (**e**) Sorghum harvested area (**f**) Soybean harvested area (**g**) Maize production (**h**) Sorghum production (**i**) Soybean production for the period 1968–2016. The thick black line in each chart depicts regional statistics across all the states.

The global drivers of agricultural production and their variability include technology, genetics, climate, soil, field management practices and associated decisions such as fertilizer applications, tillage and crop hybrid selection, irrigation management, row spacing, panting date and depth, population density, etc. A significant portion of the advances in agricultural production is a result of technological advances in genetics, agronomic and resource use practices^1–5^. For example, the sharp yield improvements in the cereal grain yields in the U.S. Great Plains region (Fig. 1) are attributed to the development and adoption of such technologies^6^ and management practices such as earlier planting dates that allow adoption of longer-maturity crop hybrids^7^ that generally yield greater than short-season hybrids.

Amongst these factors, weather and climate are prominent drivers or influencers of agricultural production systems and it has been shown that recent trends in change of climate variables may be responsible for substantially affecting crop yield trends despite advances in technology and other fronts. The Fifth Assessment Report^8^ stated that the last century experienced an increase of 0.74 °C globally in air temperatures due to increased greenhouse gas emission concentrations and the period of 1983–2012 was the warmest 30-year span over the last 800 years for the Northern Hemisphere. Also, it reports evidence of increasing precipitation, especially in mid latitudes of the Northern Hemisphere with medium confidence since 1901, but high confidence after 1951. These changes have had considerable and spatially variable effects on crop production at the global scale^9–14^, resulting in significant yield and economic losses as well as associated challenges in terms of meeting the feed, fiber, and food production of growing human population. On the contrary, there are studies^15^ that have shown that negative trends in growing season air temperatures from 1982 to 1998 that caused up to 20% yield increase in maize and soybean yields.

The aforementioned contrasting natures and magnitudes of the impacts of the climate trends are anticipated, owing to the large scale agro-climatic variations (climatological growing period length, growing degree days, rainfall patterns, and prevalence of different crop varieties) that occur in different regions. The complexity of these processes can be realized by emphasizing that the climatic factors governing crop growth and development are subject to change in both direction and magnitude spatially. Moreover, even similar trends in climate factors can result in dissimilar impacts on crop performance, which can also vary between regions. These impacts are much likely to differ among various crop species as well. As a consequence, the investigation of the climate’s impacts on crop yields requires analyses at finer scales to discern the differential spatial responses of crops to climate variability.

The U.S. Great Plains region is crucial for quantification of spatially variable impacts of historical climate onto crop yields, because of its vast geographical extent and substantial spatial and temporal variation in environmental and crop developmental factors^16,17^ and region’s significant contribution to national agricultural production and economy. The U.S. Great Plains region we consider it in this study encompasses nine states and contributes to 46%, 89% and 36% of the national maize (*Zea mays* L.), sorghum (*Sorghum bicolor*), and soybean (*Glycine max*) production and growing a significant number of other crop varieties. Moreover, this region is home to the Ogallala aquifer, which is the major source of irrigation in conjunction with surface water and is one of the largest underground aquifers in the world. This provides a unique opportunity to investigate if the climate impacts on crop yields are a function of irrigation, which is not explicitly and sufficiently reported in the literature, especially quantitatively. In the light of its contribution in the national food production and the climate variability that the region is subjected to, there are several scientific and difficult questions that need to be addressed to provide insight and scientific information and data to policy and decision makers and resource planners and managers at the national, state, and local levels for enhancing the scientific understandings of climate vs. agricultural production dynamics, which can lead to enhancements of agricultural production practices in response to changing climatic conditions:

1. *How much is the overall variability in crop yields in the region and what are the geographical patterns associated with the climate variability?*
2. *What fraction of the overall crop yield variability is explained by climate, i.e. air temperature and precipitation (climate-explained yield variability, or CEYV) and the geographic patterns in CEYV?*
3. *What climate factor (air temperature or precipitation or both) is dominant in explaining crop yield variability spatially and what is the proportion of regional food production that it affects?*
4. *What is the sensitivity of crop yields to a fixed rate change in air temperature and precipitation and how does this sensitivity vary across the region?*
5. *What are the actual climate-induced yield gains/losses for each crop across the region?*
6. *What is the role of climate-induced yield trends relative to the overall observed yield trends?*
7. *How do all the above indices differ among irrigated and non-irrigated crops and by what magnitudes?*

This manuscript analyses extensive datasets to answer the above questions for maize, sorghum, and soybean crops in the Great Plains region during the period 1968–2013. In answering the above scientific questions, this manuscript serves to potentially enhance our knowledge on climate-crop performance interactions substantially in the light of recent work in the literature. First, this manuscript focuses on three important crops for the Great Plains region, namely maize, sorghum, and soybean. While the past studies explore maize yield variability, followed by soybean^18^, sorghum has not received enough attention in this direction. It is often generally stated that sorghum is more resilient to environmental stresses than its other C_4_ counterpart, maize. By including sorghum in our study, we aim to seek observational/empirical evidence to test this prevalent claim. Moreover, sorghum contrastingly differs from maize and soybean in terms of management and technological (genetic) advancements and demonstrates slower rates of yield improvement over the course of recent decades. In fact, sorghum has been experiencing declines in planting and harvested area as well as production in the Great Plains region [Fig. 1(e,h)]. Thus, it is of crucial importance to investigate the climate impacts in influencing sorghum yields to generate insights into this slowly fading crop in terms of cultivated area. Secondly, our study addresses determination of actual historical climate impacts in terms of crop yield/production (scientific question 5) by quantifying sensitivity magnitudes of crop yields to unit changes in temperature and precipitation (scientific question 4). In addition, role of climate-induced yield trends relative to the overall observed yield trends was also evaluated by quantifying time equivalent of temperature and precipitation impacts on crop yields (scientific question 6). Addressal of these questions is critical as there is limited scientific knowledge on these aspects. A greater emphasis has been placed in the literature to determining the changes in crop yield variability and the climate contribution to influence this variability (scientific questions 1, 2 and 3). For example, Leng^19,20^ reports changes in county-level crop yield variability and the varying strength of temperature vs. yield relationship during 1980–2010 for maize in the United States. Finally, our study assesses climate impacts during 1968–2013 (46 years), which is longer than the past studies (30 years). This is particularly important in the context of determining differences in irrigated and non-irrigated crop yields, as irrigation practices in the region became prevalent in the 1960’s; thus, studies that use time period after substantial irrigation development occurred may not be able to effectively capture and evaluate the differences between irrigated and non-irrigated crop yields in relation to climate impacts. Overall, our study encompasses addressal of the fundamental of the questions such as characterizing yield variability and climate trends to more complex ones as the climate-influenced crop yield gain/loss within a single comprehensive framework, which in itself is the novelty of our work.

## Results

### Variability and Trends in Crop Yield

In this section, we attempt to answer our first scientific question, that is where and by what magnitude have the crop yields (pooled, irrigated and non-irrigated) varied inter-annually across counties during 1968–2013. To represent these changes, we used the coefficient of variation (CV), which is yield variability normalized by mean yields as a measure.

**Figure 2:**
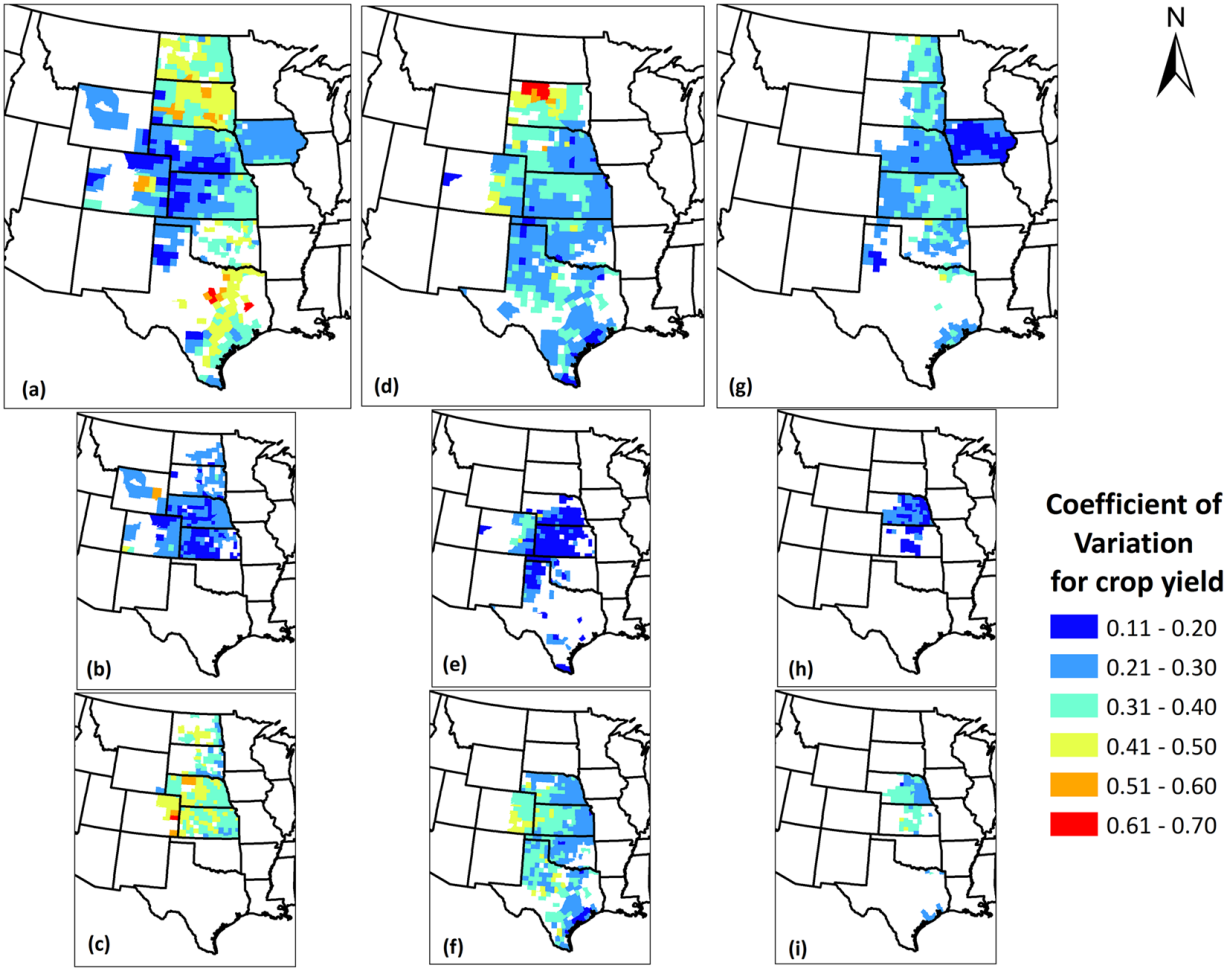
Coefficient of Variation for (**a**) Maize (**b**) Irrigated maize (**c**) Non-irrigated maize (**d**) Sorghum (**e**) Irrigated sorghum (**f**) Non-irrigated sorghum (**g**) Soybean (**h**) Irrigated soybean; (**i**) Non-irrigated soybean yields in the Great Plains counties over the period 1968–2013. Only those counties were shown which had at least 50% of the total data records. We created the maps using ESRI ArcMap 10.4.1 software http://desktop.arcgis.com/en/arcmap/.

Figure 2 presents the inter-annual variability (CV) of crop yields for counties studied in the region. We focused on the counties where at least 50% (23 years) of crop yield data were available. For maize, substantial variability existed across counties, where Nebraska, western Kansas, Texas panhandle, eastern Colorado, and Iowa had relatively lower variability than counties in North Dakota, South Dakota, Oklahoma, and central Texas. For sorghum, relatively higher CV magnitudes, which represent greater relative variability, were found in North Dakota counties than the remainder of the region. However, the region average variability was comparable to that of maize. Soybean exhibited the lowest magnitudes of CV among all three crops (Table 1), with Iowa showing the least variability. Over the last 4.5 decades, the region had a regional average variability (standard deviation, SD) of 1.8, 1.0, and 0.6 tons/ha in maize, sorghum and soybean yields, respectively, which translate to 29, 28, and 26% of regional average yields of 6.2, 3.4 and 2.2 tons/ha, for the same crops, respectively (Table 1).

**Table 1:** Regional magnitudes of the metrics employed in the study for all three crops.

Metric	Unit	Maize			Sorghum			Soybean		
		Overall	Irrigated	Non-irrigated	Overall	Irrigated	Non-irrigated	Overall	Irrigated	Non-irrigated
Mean yield	tons/ha	6.2	9.0	4.6	3.4	5.1	3.0	2.2	3.2	2.1
Yield SD	tons/ha	1.8	2.0	1.7	1.0	0.9	0.9	0.6	0.6	0.6
Yield CV	unitless	0.31	0.22	0.38	0.29	0.18	0.31	0.27	0.19	0.31
Yield Trend	kg/ha/yr	110.93	128.34	86.77	25.07	−5.95	30.93	29.14	44.15	28.11
T trend	°C/decade	−0.05	−0.02	−0.04	0.09	0.26	0.10	−0.02	−0.11	−0.03
P trend	mm/decade	6.00	4.98	3.76	1.65	−0.02	2.74	5.37	0.08	0.19
CEYV	%	18	13	40	23	21	26	23	12	34
T sensitivity	kg/ha/°C	−348	40	−374	−40	−367	−113	−45	96	−70
P sensitivity	kg/ha/10 mm	23	2	77	29	8	34	12	4	26
T sensitivity relative to mean yield	%	−4.84	0.56	−5.21	−1.11	−10.12	−3.11	−1.75	4.02	−2.94
P sensitivity relative to mean yield	%	0.33	0.02	1.07	0.81	0.23	0.93	0.52	0.15	1.07
T-induced yield impacts	kg/ha	105	67	59	−69	−405	−85	−11	−46	10
P-induced yield impacts	kg/ha	112	8	168	58	−5	47	33	25	59
T-induced yield impacts relative to mean yield	%	1.59	0.73	1.19	−2.16	−8.61	−3.68	−0.51	−1.50	0.41
P-induced yield impacts relative to mean yield	%	1.96	0.09	3.42	1.61	−0.07	1.33	1.45	0.82	2.63
Climate-induced yield impacts relative to mean yield	%	3.55	0.82	4.61	−0.55	−8.69	−2.35	0.94	−0.68	3.04
Role of T in overall yield trend	%	2.13	1.25	1.26	−15.79	31.83	20.35	−1.10	−2.30	0.78
Role of P in overall yield trend	%	1.97	0.15	4.30	3.25	2.25	−15.19	3.33	1.56	4.21
Time equivalent of role of T in overall yield trend	years	47	80	79	−6	3	5	−91	−43	128
Time equivalent of role of P in overall yield trend	years	51	678	23	31	44	−7	30	64	24

**Figure 3:**
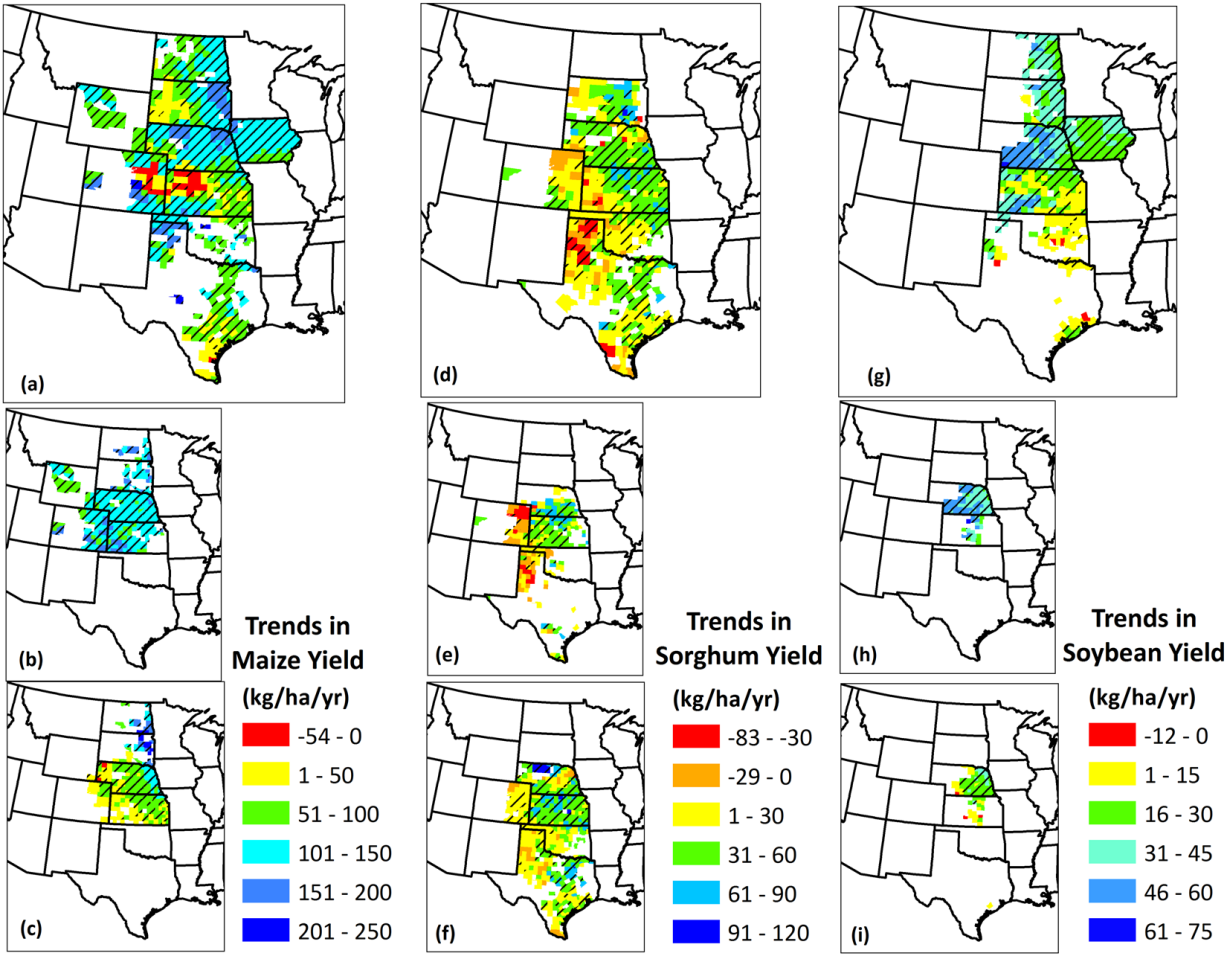
Trends in (**a**) Maize; (**b**) Irrigated maize; (**c**) Non-irrigated maize; (**d**) Sorghum; (**e**) Irrigated sorghum; (**f**) Non-irrigated sorghum; (**g**) Soybean; (**h**) Irrigated soybean; (**i**) Non-irrigated soybean yields in the Great Plains counties over the period 1968–2013. Trends are represented in kg ha^−1^ yr^−1^. Counties with striped lines indicate that the trend in the county was statistically significant to 95% confidence level. Only those counties were shown which had at least 50% of the total data records. We created the maps using ESRI ArcMap 10.4.1 software http://desktop.arcgis.com/en/arcmap/.

In addition to analyzing inter-annual variability, it is crucial to investigate the trends in crop yields to identify regions where the technological and management improvements have been adopted efficiently and others where challenges have hampered yield growth. Trends in grain yield during 1968–2013 on a county scale are presented in Fig. 3. Some of the highest maize yield increasing trends were found in eastern South Dakota, northern Nebraska, and Texas Panhandle with the highest magnitudes for Hooker County, Nebraska (261 kg/ha/yr). On the contrary, there were counties with decreasing maize yield trends that were concentrated in northwestern Kansas and Eastern Colorado, among which the most negative trend was in Kiowa County, Colorado (−54 kg/ha/yr). Over the study period, the region showed an average increasing maize yield trend of 98 kg/ha/yr, and regional average decreasing trend of 31 kg/ha/yr. Sorghum had a regional increasing trend of 34 kg/ha/yr, and a decreasing trend of 24 kg/ha/yr. Similar to maize yield trends, high geographic variability exists for sorghum yields where the highest increasing trends were in South Dakota and Nebraska with the maximum positive trend in Aurora County, South Dakota (101 kg/ha/yr). Decreasing trends were primarily concentrated in Northern Texas and Southwest Kansas counties (water-limiting areas) and the greatest negative trend was in Hansford County, Texas (83 kg/ha/yr). Lastly, soybean yields, regionally, increased at a rate of 27 kg/ha/yr, while the decreasing trends (only observed in six counties in Oklahoma and Texas) averaged at 3 kg/ha/yr. Higher soybean yield increasing trends were in North Dakota, South Dakota, Nebraska (the highest) and Iowa among which the highest magnitude was in Dundy County, Nebraska (69 kg/ha/yr). The highest negative soybean yield trend was in Leflore County, Oklahoma (6 kg/ha/yr).

Irrigated and non-irrigated crop yields were subjected to similar analyses and differences were found among them with respect to yield variability. For all three crops, it was observed that the variability in yields was considerably greater for non-irrigated crops than the irrigated crops. Visual differences in the maps in Fig. 3(b),(c),(e),(f),(h) and (i) support this observation, where lower magnitudes of CV (darker blue) were found in the irrigated crops than non-irrigated crops, for the same county. It should be noted that the National Agricultural Statistics Service (NASS) reports irrigated and non-irrigated crop yields for counties which have considerable irrigated acreage and not for all cultivated region. However, the overall crop yields [Fig. 3(a),(d),(f)] include all the cropped area irrespective of irrigation practices. When averaged across the same counties, the variability (CV) in non-irrigated yields was 77% (maize), 69% (sorghum) and 63% (soybean) greater than that in irrigated yields, which implies that irrigated systems have been producing more consistent yields. Irrigated yields are on an average 100% (maize), 69% (sorghum) and 53% (soybean) greater than non-irrigated yields. Crop yield trends computed under irrigated and non-irrigated settings also differed, with irrigated maize and soybean yields increasing at a greater rate than non-irrigated yields, but at a slower pace for sorghum. Specifically, irrigated maize and soybean yields increased at a rate of 60% and 85%, respectively, greater than the rate of increase of non-irrigated yields. For sorghum, however, irrigated yields increased at a rate that was lower by 17% than the non-irrigated yields. This is attributed to the prevalence of some counties in Eastern Colorado and Texas Panhandle, which had negative irrigated sorghum yield trends and positive non-irrigated sorghum yield trends, reducing the difference between irrigated and non-irrigated sorghum yields.

### Climate trends

**Figure 4:**
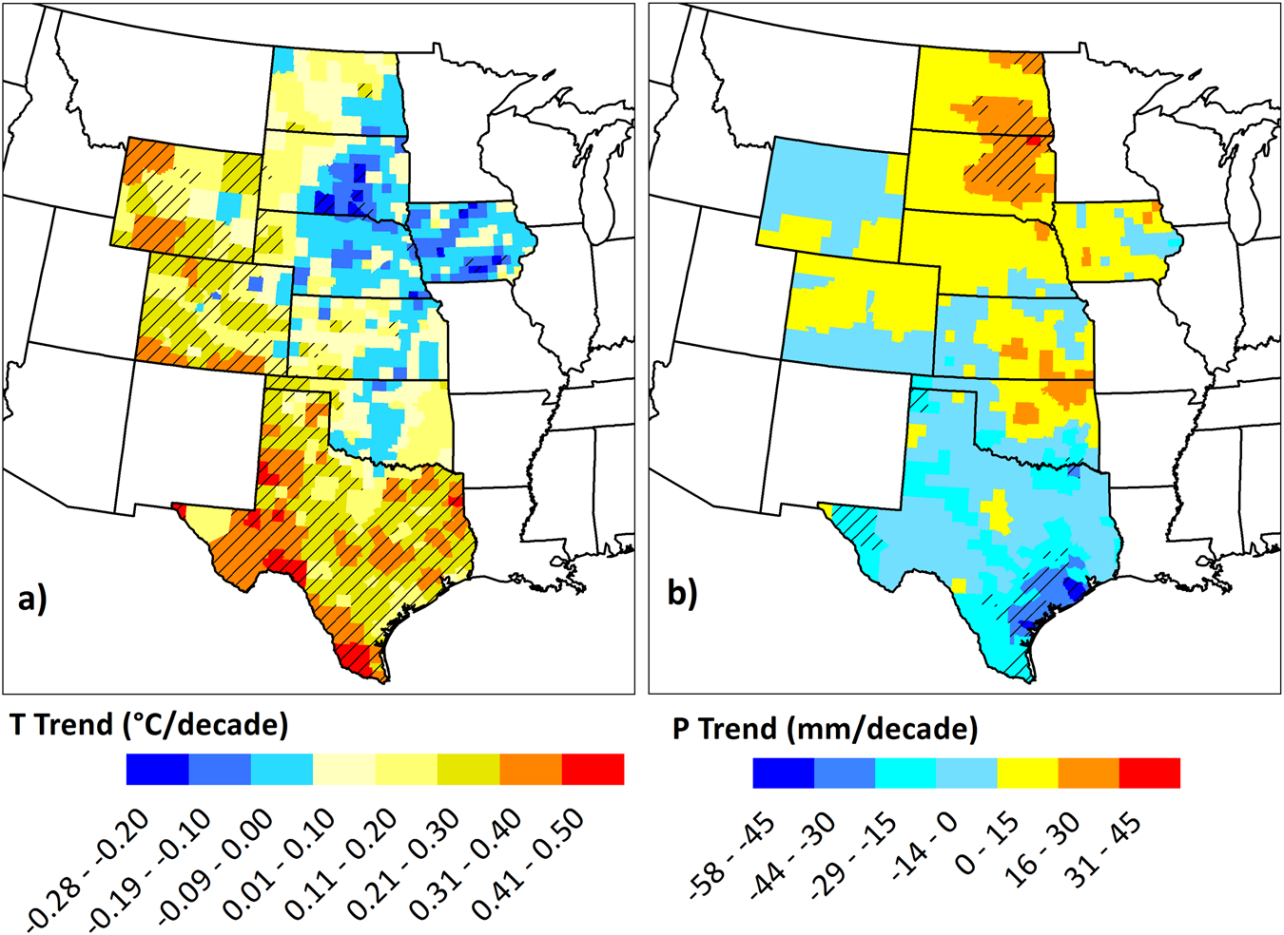
Trends in growing season (May 1^st^ to September 30^th^) (**a**) Average Air Temperature (**b**) Precipitation totals in the Great Plains counties over the period 1968–2013. Temperature trends are represented in °C decade^−1^ and precipitation trends are represented in mm decade^−1^. Counties with striped lines indicate that the trend in the county was statistically significant to 95% confidence level. We created the maps using ESRI ArcMap 10.4.1 software http://desktop.arcgis.com/en/arcmap/.

The trends that were observed in average air temperature and total precipitation during the growing season (1^st^ May-30^th^ September) for 1968–2013 period for each county of the Great Plains region are presented in Fig. 4. It is evident that these trends, like crop yield trends, are characterized by high geographic variability, in both nature and magnitude of the trend. Trends in growing season average temperature varied from a negative (cooling) trend of 0.28 °C/decade in Audubon County, Iowa to a positive (warming) trend of 0.45 °C/decade in Midland County, Texas. The southern and western parts of the region, which included counties in Texas, Colorado, and Wyoming, western parts of Dakotas, Nebraska, Kansas, and western and eastern Oklahoma generally showed increasing trends. On the contrary, the northern, central and eastern parts, consisting of counties in the eastern Dakotas, Nebraska, Kansas, Central Oklahoma, and Iowa showed negative trends. The regional average of warming trends (for 578 counties that show warming trends) was 0.18 °C/decade, while the regional average of cooling trends (for 256 counties) was −0.08 °C/decade. Of these trends, 51% and 7% county-level trends were statistically significant (*p* < 0.05).

Similar to the trends in average temperature, high spatial variability was observed in the precipitation trends across the Great Plains. The highest positive trend (increasing trend or becoming wetter) observed was 30 mm/decade in Marshall County, South Dakota, while the highest negative trend (decreasing trends or becoming drier) was −58 mm/decade in Brazoria County, Texas. For precipitation, 424 counties showed increasing trends, while 410 counties showed decreasing trends, as opposed to temperature trends, where the majority of trends were increasing. The regional averages of the increasing and decreasing trends were 9 mm/decade and −12 mm/decade, respectively. In a very similar manner as the trends in temperature, the geographic distribution of the precipitation trends can be considered of increasing/decreasing trends separated by an imaginary line through northwest edge to southeast edge. In other words, the regions which show warming trends are roughly the same as those show drying trends (decreasing precipitation trends), and the areas with cooling trends (decreasing temperature trends) the ones that show wetting trends (increasing precipitation trends). The proportion of counties where significant trends were found were considerably lower as compared to temperature trends and 8% and 8.5% of the counties showing increasing and decreasing trends, respectively.

These climate trends, whether significant or insignificant statistically, can still have significant implications on agricultural crop production by affecting yields, irrigation management and crop water demand, risk of diseases/pests, soil temperature, growing season length, etc., and other crop, and soil management practices and hence demand appropriate consideration while investigating climate change impacts on crop yields.

### Climate Explained Yield Variability

The climate explained yield variability (CEYV), represented by the R^2^ of the county-specific models of yield residuals and climate residuals indicates the degree of variability in crop yields that can be explained by climate variability. At what locations and by what degree the climate variability was responsible for variability in crop yields was highly site-specific, crop-specific, and irrigation class-specific (i.e., irrigated or non-irrigated). We present the results of these analyses for each crop, which further include the pooled general crop datasets and irrigation-based classification (irrigated and non-irrigated).

**Figure 5:**
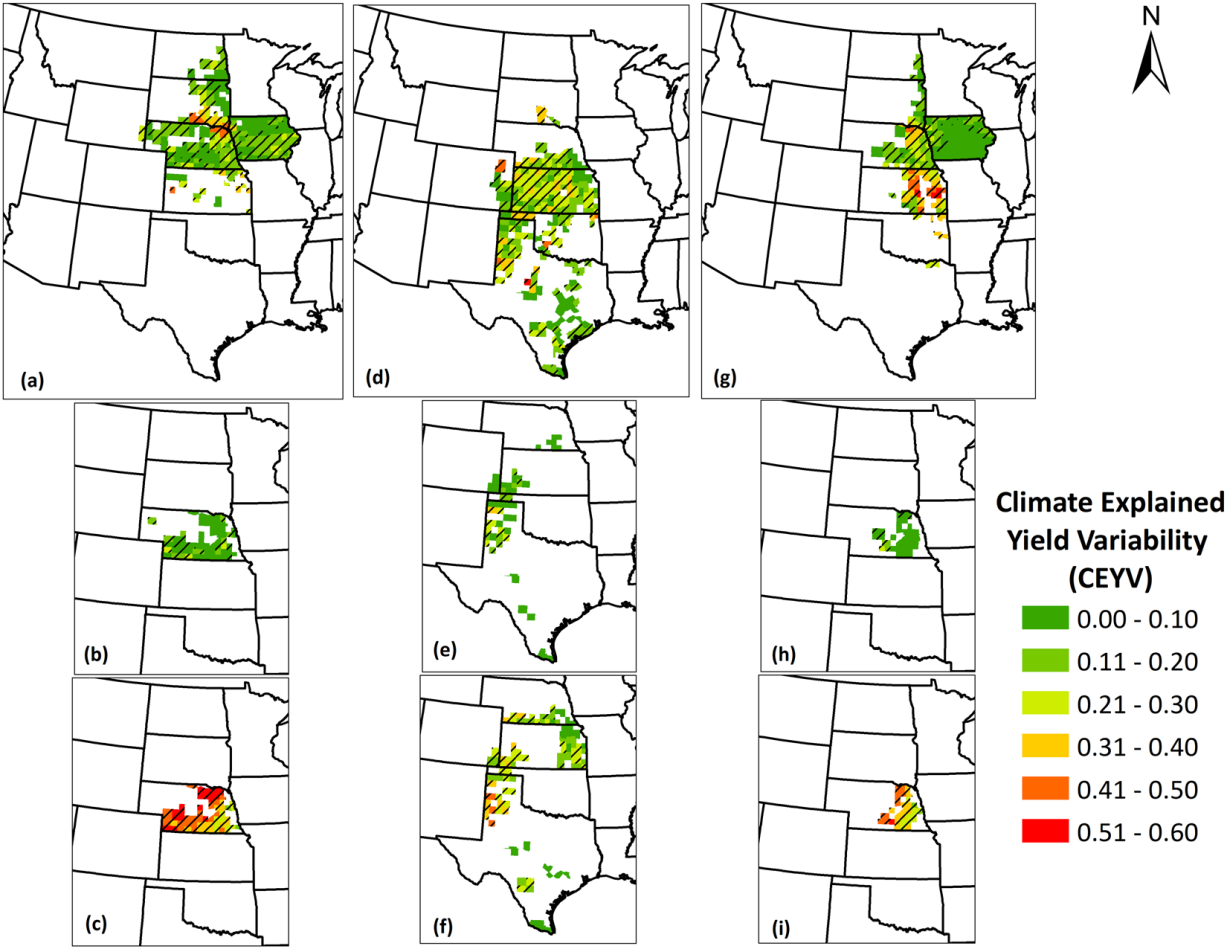
Climate explained crop yield variability (CEYV) for in (**a**) Maize; (**b**) Irrigated maize; (**c**) Non-irrigated maize; (**d**) Sorghum; (**e**) Irrigated sorghum; (**f**) Non-irrigated sorghum; (**g**) Soybean; (**h**) Irrigated soybean; (**i**) Non-irrigated soybean yields in the Great Plains counties over the period 1968–2013. The values range from 0 to 1. A value of 1 implies that complete variability in crop yields was explained by climate variability and likewise a value of 0.2 implies that 20% of the variability in crop yields was explained by climate variability. Counties with striped lines indicate that at least one climate factor significantly explained crop yield variability in that county at 95% confidence level. Regions with no data indicate that the crop yield dataset for a particular county was not complete. We created the maps using ESRI ArcMap 10.4.1 software http://desktop.arcgis.com/en/arcmap/.

The inter-annual variations in crop yields that can be explained by inter-annual variations in climate are presented in Fig. 5, which shows information for counties with complete data record for grain yield (246 counties for maize, 242 for sorghum, and 225 for soybean). The climate explained a wide range of variation in crop yields, ranging from no explanation to 52% variability explained across counties. Averaged across the counties where either temperature or precipitation had statistically significant impacts, 18, 23, and 23% of the variability in maize, sorghum, and soybean were explained by climate variability. For maize, the counties along the boundary adjoining northeast Nebraska and southeast South Dakota primarily showed relatively higher (>20%) explanation of maize yield variation by climate variation. The soybean CEYV is highly variable most of the counties show small (<10%) and moderate (10–20%) CEYV, especially in Kansas and Texas Panhandle. For soybean yields, most of the highest CEYV magnitudes were found in Kansas and northeast Nebraska. Most of the counties in Iowa showed small to medium magnitudes of CEYV (0–20%).

Irrigated and non-irrigated crops differed in the degree of explanation of their yield variability by climate variability. The contrasting representation of the CEYV in irrigated and non-irrigated crops in Fig. 5 evidently signifies these differences. These differences can be better highlighted by averaging and comparing CEYV magnitudes across the mutual counties (possess data for both irrigated and non-irrigated CEYV). The analyses revealed that irrigated and non-irrigated maize showed CEYV of 8% and 41%, respectively, with the latter being 436% higher than the former. Similarly, CEYV in non-irrigated sorghum (23%) and non-irrigated soybean (35%) yield was 160% and 728% higher than irrigated sorghum (9%) and irrigated soybean (4%). This suggests that the irrigated cropping systems are relatively robust to encounter climate variability than their non-irrigated counterparts in that irrigation practices are effective drought-mitigation strategy in term of maintaining grain yield and non-irrigated yields are substantially more vulnerable to climate impact(s) on yields.

### Climatic Driver(s) of Crop Yield Variability

**Figure 6:**
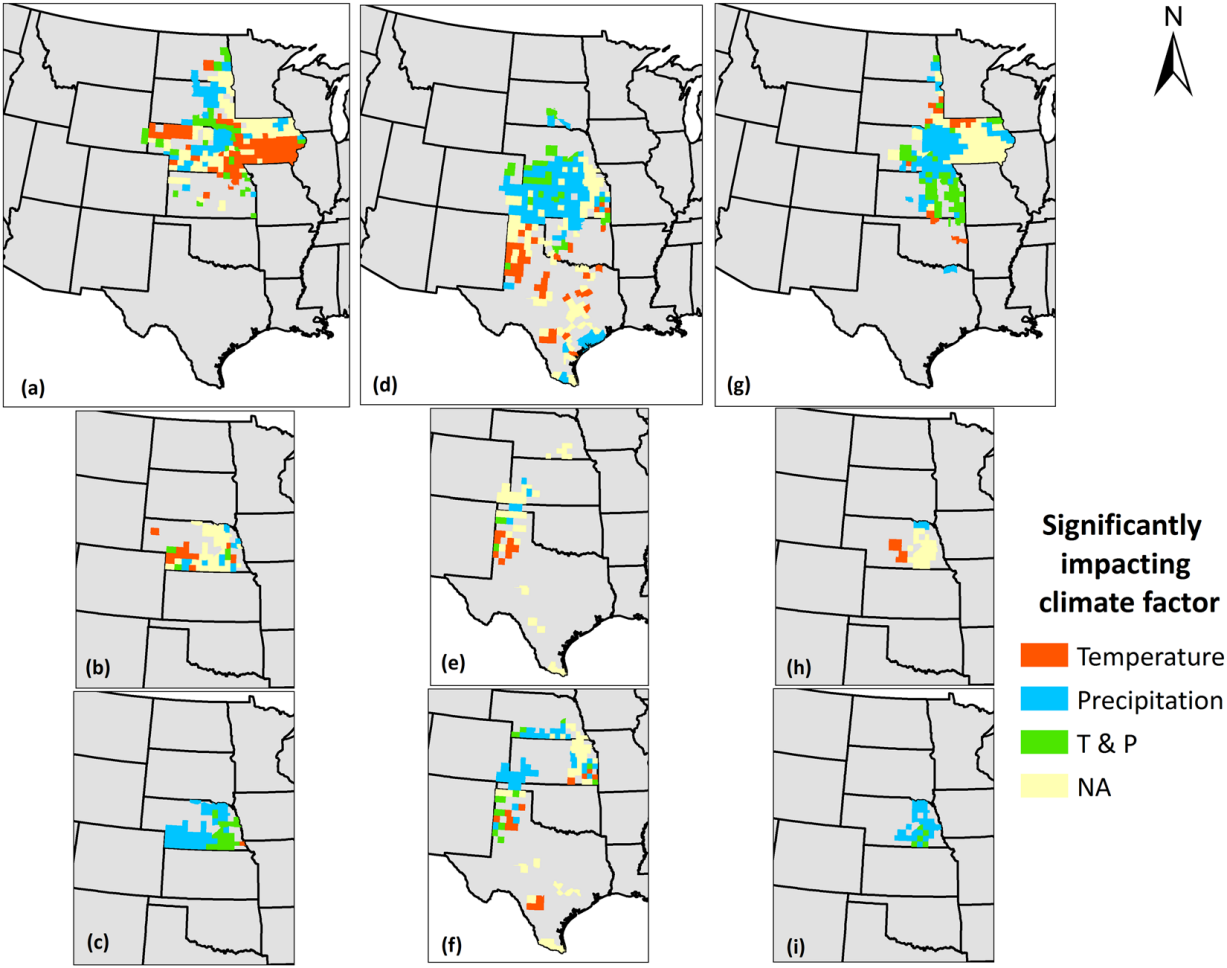
Dominant climate factor (significant) responsible for explaining crop yield variability in (**a**) Maize; (**b**) Irrigated maize; (**c**) Non-irrigated maize; (**d**) Sorghum; (**e**) Irrigated sorghum; (**f**) Non-irrigated sorghum; (**g**) Soybean; (**h**) Irrigated soybean; (**i**) Non-irrigated soybean yields in the Great Plains counties over the period 1968–2013. Four outcomes including temperature only, precipitation only, both temperature and precipitation, and NA are possible. NA indicates that no climate factor was able to explain crop yield variability at 90% confidence level. We created the maps using ESRI ArcMap 10.4.1 software http://desktop.arcgis.com/en/arcmap/.

The observed climate-driven crop yield variability has to be further characterized to understand what specific factor(s) (temperature, precipitation, or both) play a dominant role in influencing crop yield variability. Spatial differences in these drivers of crop yields as well as the crop-specific and irrigation-dependent controls were highlighted in this section. Our analyses revealed that considerable variability exists in the dominance of climatic factors in explaining US Great Plains maize, sorghum, and soybean yield variability (Fig. 6). Temperature alone was able to explain maize yield variability in northwest and southeast Nebraska and southern counties in Iowa while it was responsible in explaining sorghum yield variation in almost all significantly affected counties in Texas, except a few counties in the southeastern part. In contrast, temperature solely did not explain soybean yield variability, except for only a few scattered counties in Southeast South Dakota and Northwest Iowa. Precipitation, on the other hand, was able to explain maize yield variability in central and northeast South Dakota, central, southwest and northeast Nebraska. For sorghum and soybean, precipitation was responsible for explaining yield variability in considerable proportion of counties. These regions were Kansas and eastern Colorado for sorghum and eastern Nebraska, eastern Iowa and some parts of Kansas for soybean. Interestingly, and importantly, there were counties where both temperature and precipitation explained variability in crop yields such as southeast South Dakota for maize, central Nebraska and western Kansas for sorghum and Kansas for soybean yields. Lastly, there were counties where climate variability did not explain crop yield variability significantly (*p* < 0.10; counties represented in yellow color). *Leng et al*.^18^ reported dominant climate factors significantly explaining crop yield variability for maize and soybean, and included seven possible combinations of climate factors that could be assigned to a particular county, in contrast to the three factors employed in this study. Observations of spatial dependence of dominant climatic factors vividly demonstrate that influence of climate variability on crop yield variability are highly site-specific and should be addressed at finer scales, rather than statewide, national, continental, or global scales.

As seen previously, irrigated crop yield variability was explained by a different magnitude than non-irrigated crop yield variability. In addition, our analyses establishes that the dominant climate factors than explain yield variability also varies among irrigated and non-irrigated crops. Overall, it was found that the non-irrigated crop yield variability was largely explained by precipitation alone and both precipitation and temperature, whereas temperature alone explained irrigated crop yield variability primarily. For instance, temperature only explained irrigated maize yield variability in southwest Nebraska, irrigated sorghum yield variability in Texas Panhandle, and irrigated soybean yield variability in central Nebraska, whereas counties where precipitation only or temperature and precipitation explained irrigated crop yields were minimal. The exact was opposite for non-irrigated crop yields, where temperature and precipitation explained maize yield variability in the eastern Nebraska and precipitation only in the rest of the state, precipitation only explained sorghum yield variability in southern Nebraska, southwest and eastern Nebraska and southeast Colorado, and finally precipitation only primarily explained soybean yield variability.

**Figure 7:**
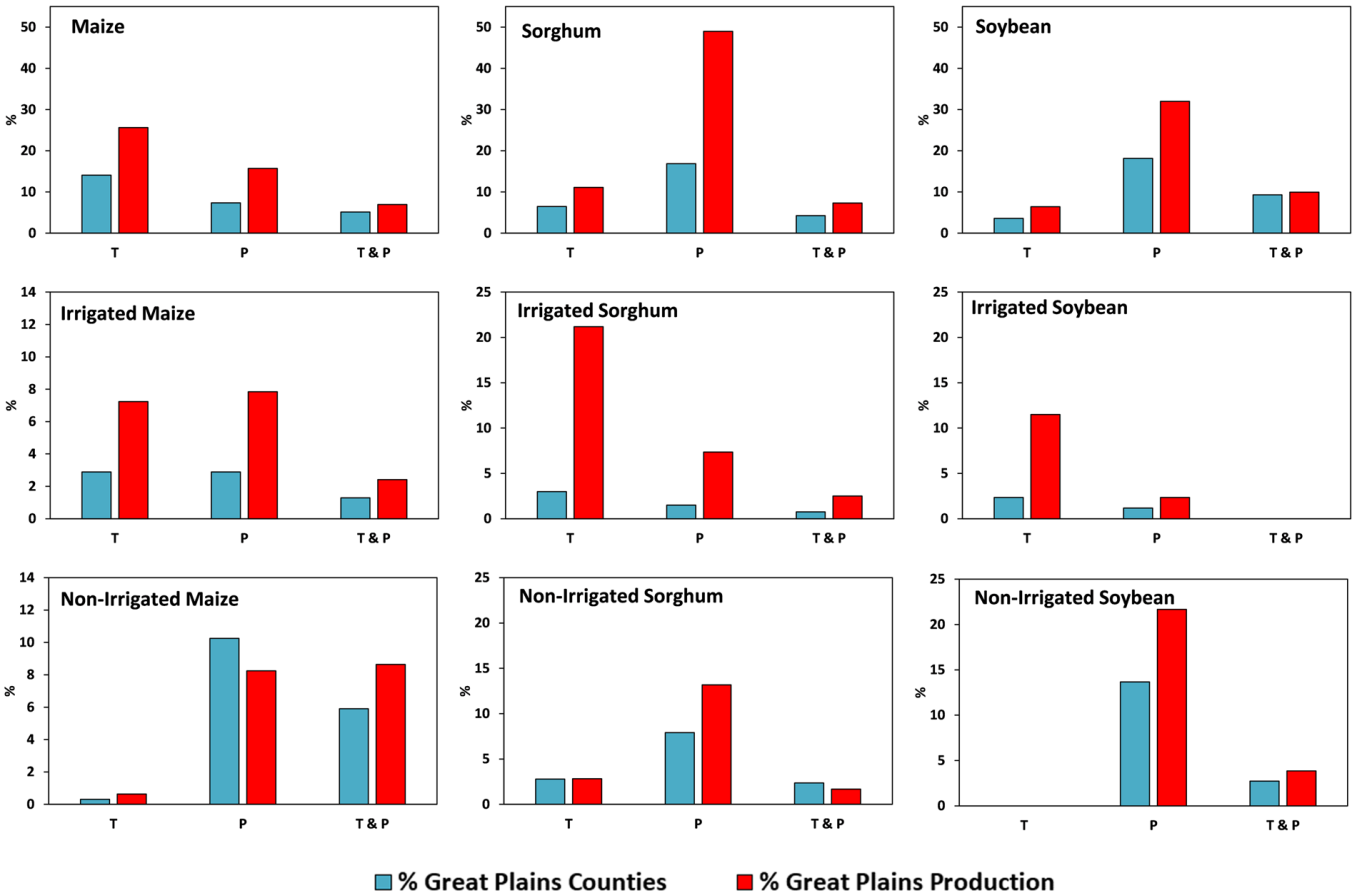
Proportion of Great Plains counties and crop production for maize, sorghum, soybean yields (pooled, irrigated and non-irrigated) where temperature, precipitation, or both were the dominant climate factor towards explaining crop yield variability. Blue bars indicate proportion of Great Plains counties for which a specific climate factor was dominant and red bars indicate proportion of Great Plains production for which a specific climate factor was dominant.

We also analyzed which dominant climate factor explained crop yield variability in what percentage of counties, and further how much these counties contribute to the overall Great Plains crop production. These analyses would help us interpret the relative potential impacts of climate factors to the regional food security and eventually, the national food security. Figure 7 presents the percentage of crop growing counties and their contribution to the Great Plains production for each climate factor that dominantly explains crop yield variability. Temperature alone explained yield variability in 14%, 6% and 4% of the maize, sorghum, and soybean growing counties, respectively, which contribute to 26%, 11% and 6% of the total Great Plains production. Precipitation alone, on the other hand explained 7%, 17% 18% of maize, sorghum, and soybean growing counties, which was lower for maize and higher for sorghum and soybean than what was attributed to temperature. The counties where crop yield variability was dominantly explained by precipitation alone contributed to 16%, 49% and 32% of Great Plains’ maize, sorghum and soybean production, respectively. Interestingly, the counties where precipitation explained sorghum (central and western Kansas) and soybean (eastern Nebraska) yield variability contributed to half (sorghum) and a third (soybean) of the Great Plains production, which is substantial. Lastly, temperature and precipitation combined dominantly explained yield variability in 5%, 4% and 9% of maize, sorghum, and soybean growing counties, respectively, which contributed 7%, 7% and 10% to the Great Plains total yield production. Irrigated crop yield variability is explained dominantly by temperature alone for sorghum and soybean, but equally by temperature and precipitation alone for maize. Again, for irrigated sorghum, counties where yield variability was explained by temperature alone were only 3% of irrigated sorghum counties, but they contributed to 21% of the Great Plains irrigated sorghum production. Non-irrigated crop yield variability, as stated earlier, was dominantly explained by precipitation alone for 10%, 8% and 14% of Great Plains counties growing maize, sorghum, and soybean, respectively.

### Sensitivity of Crop Yields to Climate Factors

**Figure 8:**
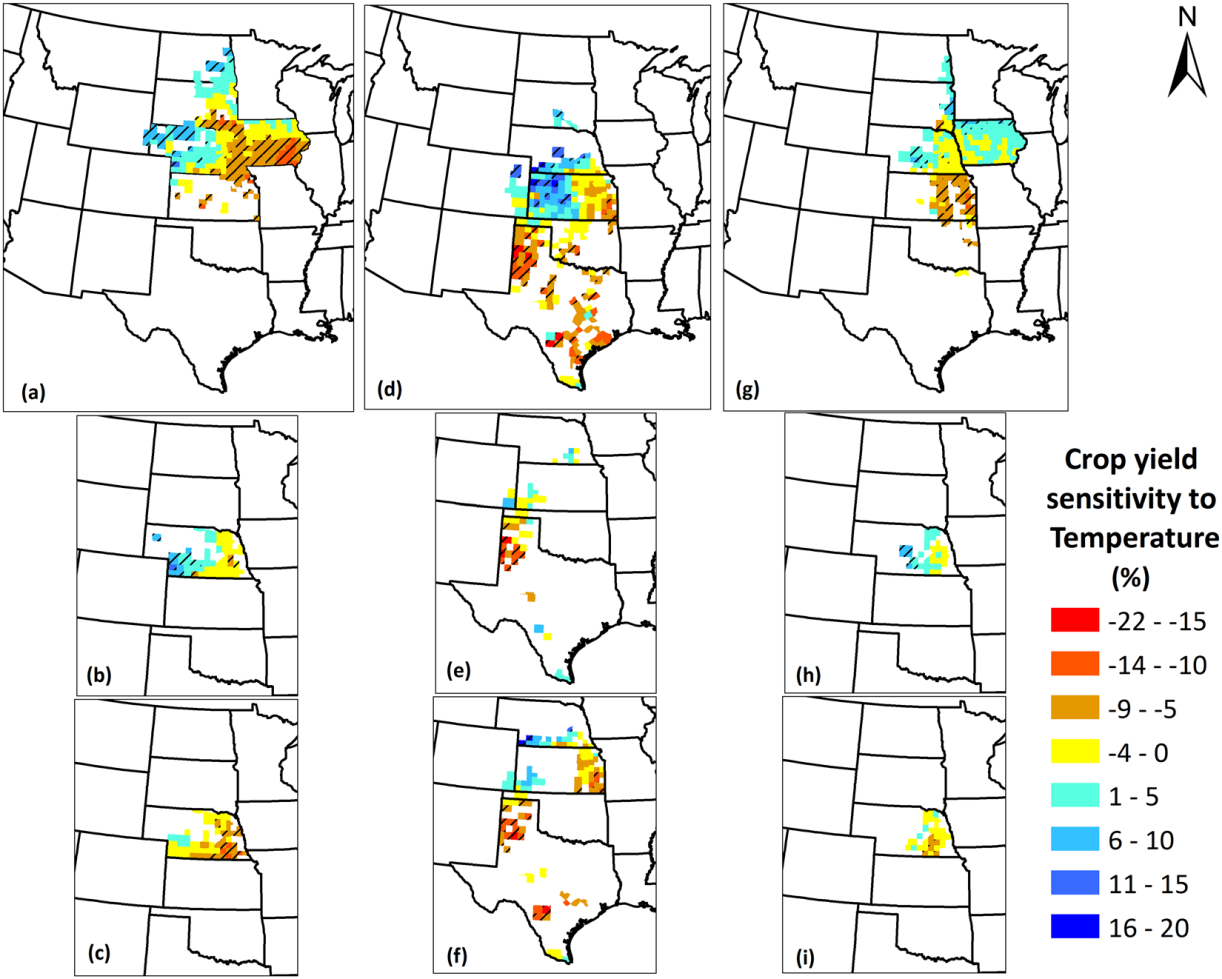
Crop yield sensitivity to temperature for (**a**) Maize; (**b**) Irrigated maize; (**c**) Non-irrigated maize; (**d**) Sorghum; (**e**) Irrigated sorghum; (**f**) Non-irrigated sorghum; (**g**) Soybean; (**h**) Irrigated soybean; (**i**) Non-irrigated soybean yields in the Great Plains counties over the period 1968–2013. The value represents change in crop yields as percentage of mean crop yields over 1968–2013 per 1 °C increase in temperature. Negative values indicate that the crop yields decrease with temperature rise and positive values indicate that they increase with temperature rise. Counties with striped lines indicate that the sensitivity magnitudes were statistically significant at 90% confidence level. We created the maps using ESRI ArcMap 10.4.1 software http://desktop.arcgis.com/en/arcmap/.

**Figure 9:**
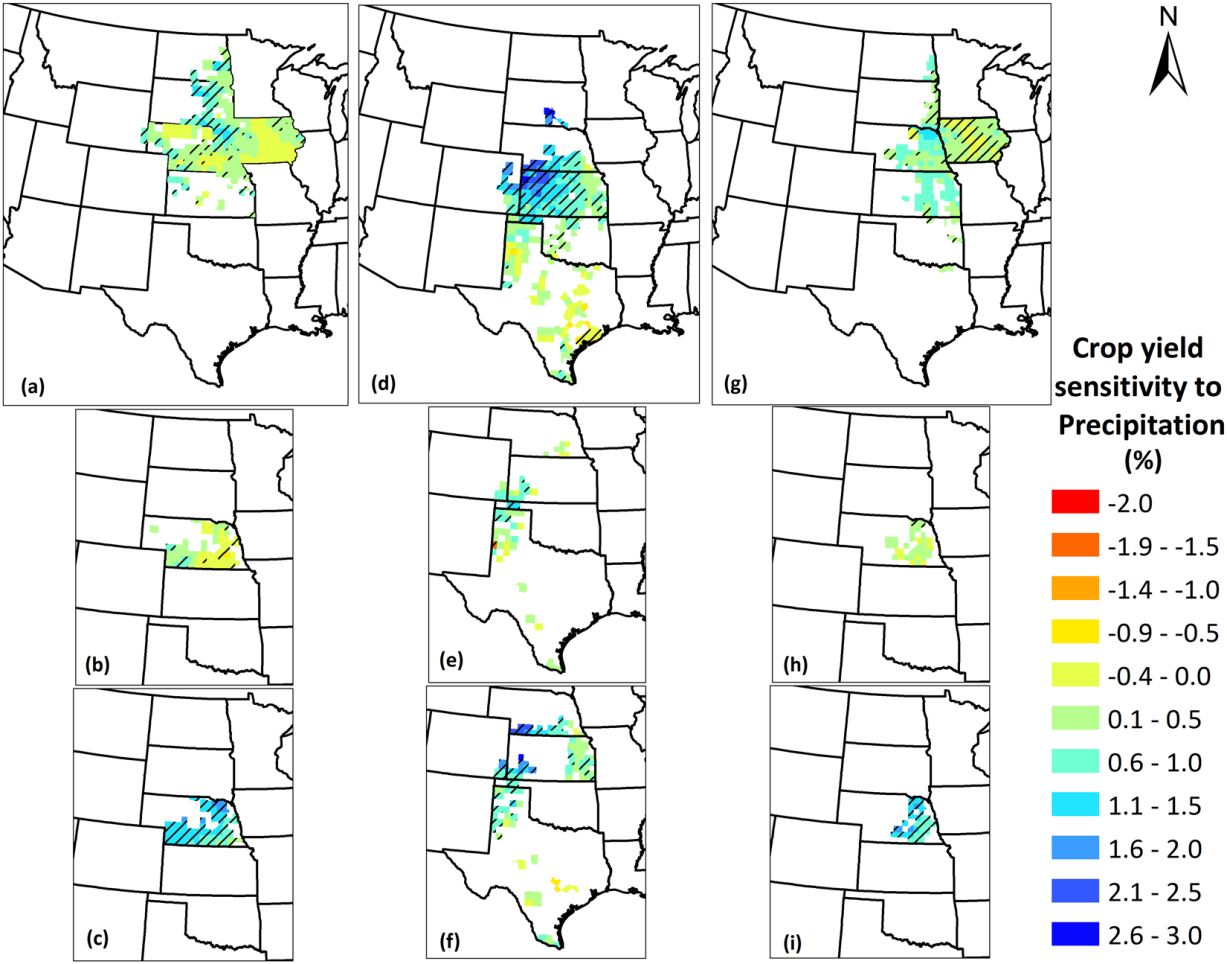
Crop yield sensitivity to precipitation for (**a**) Maize; (**b**) Irrigated maize; (**c**) Non-irrigated maize; (**d**) Sorghum; (**e**) Irrigated sorghum; (**f**) Non-irrigated sorghum; (**g**) Soybean; (**h**) Irrigated soybean; (**i**) Non-irrigated soybean yields in the Great Plains counties over the period 1968–2013. The value represents change in crop yields as percentage of mean crop yields over 1968–2013 per 10 mm increase in precipitation. Negative values indicate that the crop yields decrease with precipitation rise and positive values indicate that they increase with precipitation rise. Counties with striped lines indicate that the sensitivity magnitudes were statistically significant at 90% confidence level. We created the maps using ESRI ArcMap 10.4.1 software http://desktop.arcgis.com/en/arcmap/.

In order to estimate the spatial impacts of individual climate factors on crop yields over the study period, it is necessary to quantify the sensitivity of the crop yields to the climate factors. The sensitivity magnitudes in combination with the climate trends result in actual estimates of climate impacts on crop yields. The sensitivity metric aids to inform the change in crop yields that occur with a unit change in climate factors. In this section, we present magnitude and direction of change in crop yields (as percentage of mean crop yields over 1968–2013 period) per 1 °C increase in temperature and per 10 mm increase in precipitation. Figures 8 and 9 show the sensitivity of crop yields to growing season temperature and precipitation, respectively for the region. Temperature rise was found to negatively affect maize yields in eastern Nebraska and Iowa, with instigating up to 22% decrease in yield with 1 °C rise in temperature. However, in western Nebraska and eastern North Dakota, it positively affected maize yields, implying that temperature rise in these regions is beneficial to crop yields. For sorghum, a positive relationship existed among yields and temperature in Nebraska and western Kansas, while a negative correlation was found in Texas. Soybean crop yields were positively impacted by temperature rise in central Nebraska and eastern South Dakota, whereas they were negatively affected in Kansas. It is noteworthy that the extremes in sensitivity magnitude in either direction were found in sorghum growing regions, ranging from −21% to 17%, which are the highest among the three crops. Precipitation, on the other hand was found to primarily have a positive correlation for maize and sorghum crops, while a negative correlation did exist for soybean. Some key areas where maize yields benefit from precipitation increase are western, central, and northeastern Nebraska, northeastern Iowa, eastern South Dakota, and North Dakota. Similarly, Kansas, southern Nebraska, eastern Colorado, and western and central Oklahoma show positive correlation of sorghum yield with precipitation. Soybean, on the contrary showed both positive and negative correlations with yields, especially in Iowa. Among the three crops, sorghum yields show highest positive sensitivity to precipitation (3%).

Sensitivity of irrigated and non-irrigated crop yields to climate factors also varied among each other. Irrigated maize shows positive correlation in western Nebraska, as opposed to the negative correlation in southeastern Nebraska for non-irrigated maize. Sorghum yields show highly negative sensitivity (22%) with temperature rise for both irrigated and non-irrigated conditions in the Texas panhandle, in addition to some counties in southeastern Kansas and southern Texas, showing negative correlation for non-irrigated sorghum. Soybean yields however, show positive correlation with temperature rise in central Nebraska under irrigated conditions, and negative correlation in south-central Nebraska under non-irrigated conditions. Precipitation rise had significant positive sensitivity for almost all counties under non-irrigated conditions, which implies that all rainfed crop yields are benefitted by any increase in precipitation. For irrigated crops, relatively lesser proportion of counties show positive sensitivity, and with lower magnitudes than non-irrigated conditions, with increase in precipitation. Irrigated maize show both positive correlation (in western Nebraska) and negative correlation (in eastern Nebraska), whereas irrigated sorghum and soybean show positive correlations. On average, sensitivity (negative) to temperature of non-irrigated maize was twice that of irrigated maize, however, irrigation did not result in improved (lower) sensitivity in sorghum as the sensitivity of irrigated sorghum was 0.1 times greater than non-irrigated sorghum. Precipitation sensitivity, was higher for non-irrigated yields for all crops, and was 43 times (maize), 3.1 times (sorghum), and 3.6 times (soybean) that of irrigated yields.

The manifestation of a climate impact at a location is governed by nature (positive or negative correlation) and magnitude (percentage change in yield) of sensitivity (discussed in the section above) and the climate trends at that location (discussed earlier). There may be locations which are highly sensitive to temperature and/or precipitation, but the actual change in temperature and/or precipitation is low, or vice versa, which will have an impact on the actual effect realized at that location.

### Climate-Induced Yield Impacts

**Figure 10:**
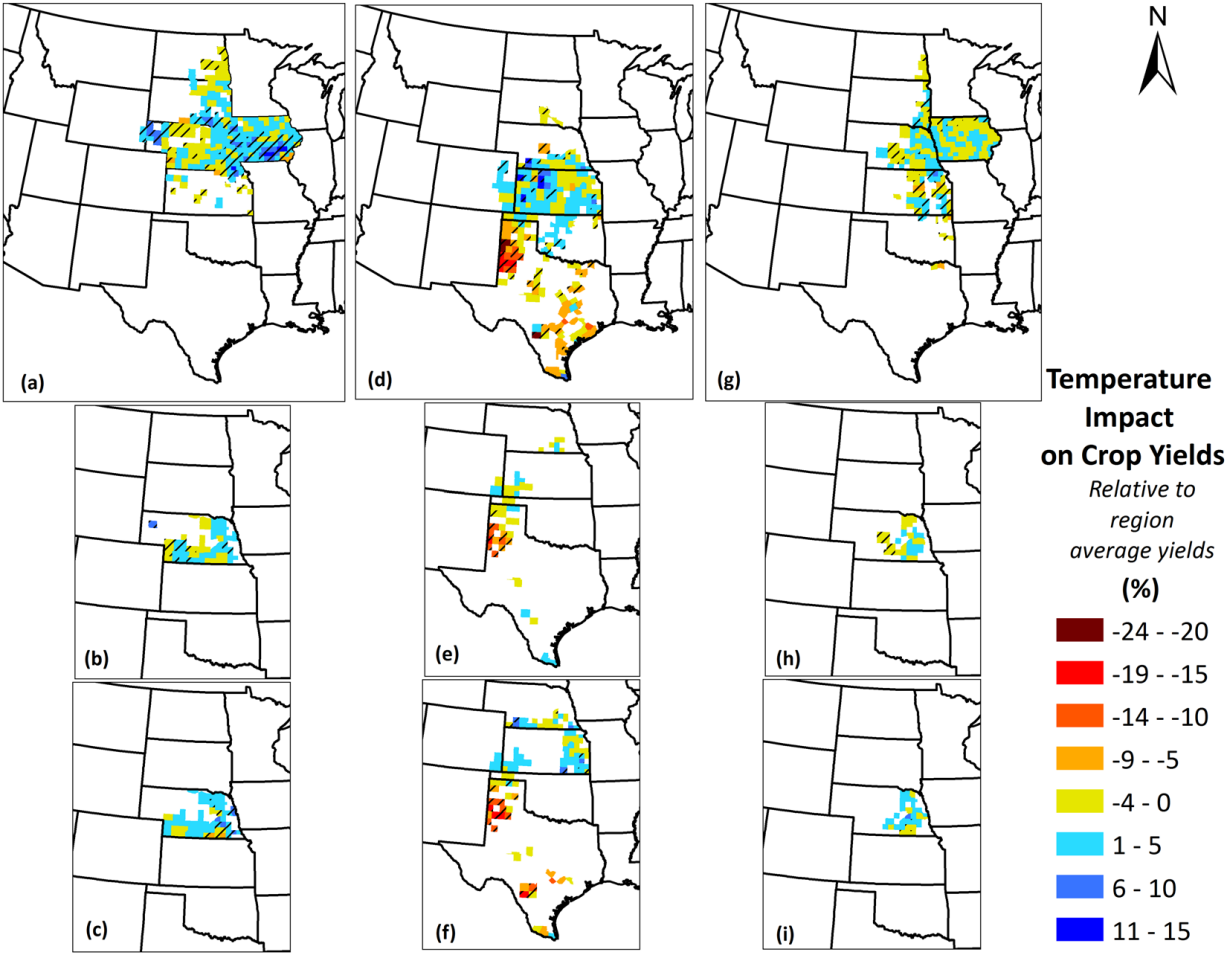
Temperature-induced crop yield impacts for (**a**) Maize; (**b**) Irrigated maize; (**c**) Non-irrigated maize; (**d**) Sorghum; (**e**) Irrigated sorghum; (**f**) Non-irrigated sorghum; (**g**) Soybean; (**h**) Irrigated soybean; (**i**) Non-irrigated soybean yields in the Great Plains counties over the period 1968–2013. Values are represented as percentage of region-average yields. A positive sign indicates that the observed trends in temperature whether increasing or decreasing, have positively impacted crop yields, while a negative sign indicates that observed trends in temperature have negatively impacted crop yields. Counties with striped lines indicate that the temperature-induced impacts were statistically significant at 90% confidence level. We created the maps using ESRI ArcMap 10.4.1 software http://desktop.arcgis.com/en/arcmap/.

**Figure 11:**
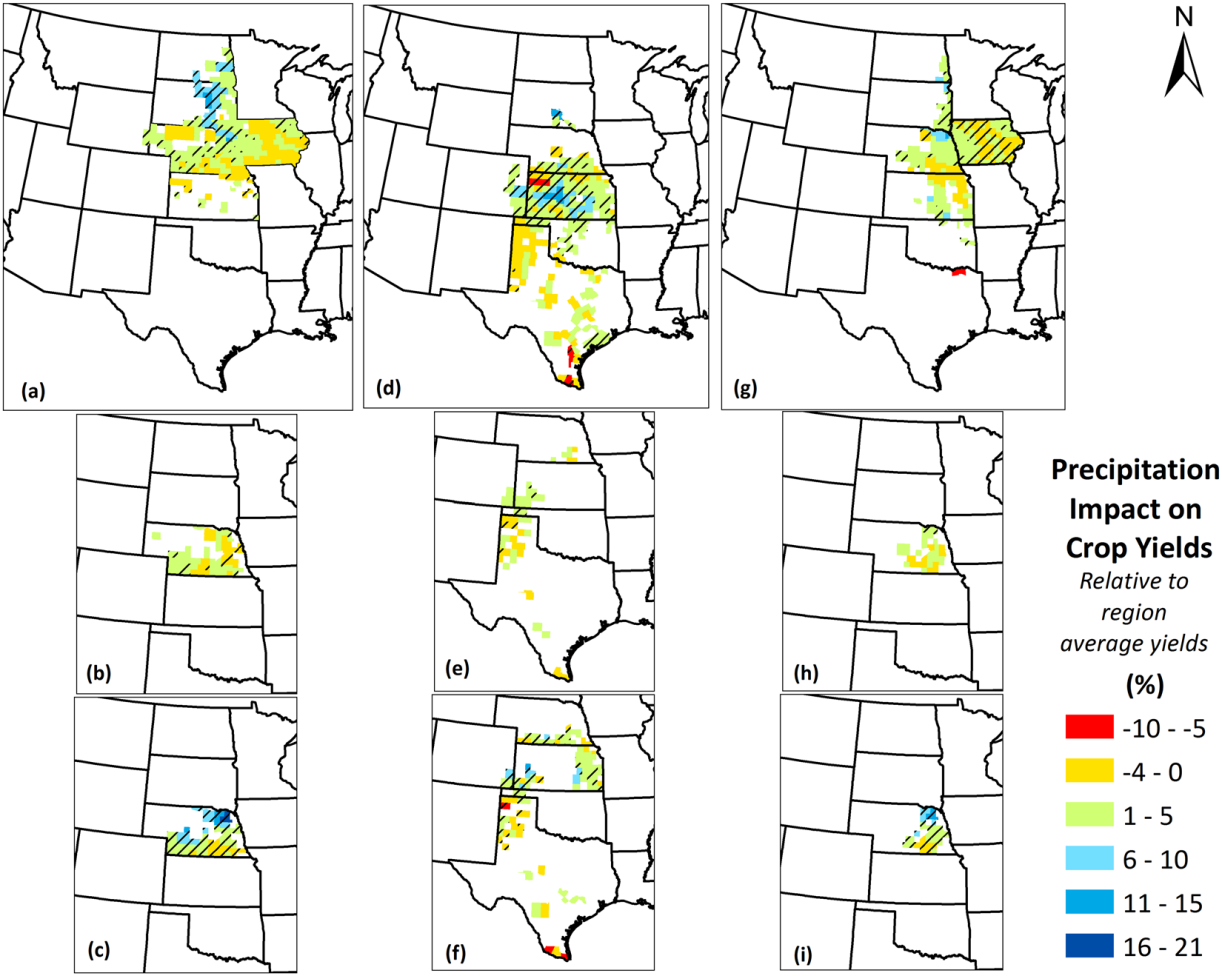
Precipitation-induced crop yield impacts for (**a**) Maize; (**b**) Irrigated maize; (**c**) Non-irrigated maize; (**d**) Sorghum; (**e**) Irrigated sorghum; (**f**) Non-irrigated sorghum; (**g**) Soybean; (**h**) Irrigated soybean; (**i**) Non-irrigated soybean yields in the Great Plains counties over the period 1968–2013. Values are represented as percentage of region-average yields. A positive sign indicates that the observed trends in precipitation whether increasing or decreasing, have positively impacted crop yields, while a negative sign indicates that observed trends in precipitation have negatively impacted crop yields. Counties with striped lines indicate that the precipitation-induced impacts were statistically significant at 90% confidence level. We created the maps using ESRI ArcMap 10.4.1 software http://desktop.arcgis.com/en/arcmap/.

This metric serves to inform the relative proportion of the climate-induced yield change in a particular county as compared to the average county-specific yield or regional average yield. This assists to visualize the degree of climate-induced yield change as percentage of average yields and realize and conclude if the climate impact in a county is substantial or negligible. The higher the percentage, the higher is the yield change due to climate with respect to the average yield obtained and vice-versa. Moreover, a positive sign indicates that the trends in temperature and precipitation, whether increasing or decreasing, have positively impacted crop yields, while a negative sign indicates that trends in temperature and precipitation, had negative impact on crop yields. For all crops, the yields were observed to be affected both positively and negatively by trends in climate. As is clear, the areas where the three crops are grown are different, especially for sorghum (shares the least common area with maize and soybean). The temperature and precipitation trends were highly spatially variable and inconsistent, and hence it is anticipated that the impacts of these trends on crop yields are spatially dependent as well. Figures 10 and 11 present the temperature and precipitation induced yield impacts for all relevant counties in the region, respectively.

In the respective crop regions, the proportion of negatively impacted counties (with temperature-induced losses) were higher than positively impacted counties (with temperature-induced gains) for soybean and sorghum, while the opposite was true for maize. Although the highest magnitudes of impacts were much higher, the regional average temperature-induced yield gains were 4.3%, 3.9%, and 2.2%, while the temperature-induced yield losses were 1.9%, 9.8%, and 2.6% for maize, sorghum and soybean, respectively. On average, temperature-induced net impacts were 1.6%, −2.2%, and −0.5% for maize, sorghum, and soybean, respectively, which imply that on average maize yields benefitted and sorghum and soybean yields suffered from the temperature changes. For maize, the regions of negative impacts were central Nebraska and North Dakota, while the rest of the region primarily observed to be positively impacted. Sorghum was found to have negative impacts in Texas counties with the Texas panhandle experiencing severe yield losses of up to 34% of the county average yields during the 46-year period. Impacts on soybean yields were located in counties in central and eastern Kansas, central Nebraska, southeastern South Dakota, and northern Iowa.

Precipitation-induced impacts were less prevalent for maize (significant impacts found in 35% lesser counties), and more prevalent for sorghum (significant impacts found for 116% additional counties) and soybean (significant impacts found for 96% additional counties). Moreover, unlike temperature impacts, the proportion of positively impacted counties was greater than that of negatively impacted counties for all the crops. In fact, majority of the impacts were positive with only 12%, 30%, and 19% of impacted counties suffering yield losses for maize, sorghum, and soybean, respectively. The regional average precipitation-induced yield gains were 5.2%, 3.4%, and 2.8%, whereas the precipitation-induced yield losses were 1.4%, 3.5%, and 1.7% for maize, sorghum, and soybean, respectively. Overall, the net impacts for the region were positive (yield gains) of 2.0%, 1.6%, and 1.4% for maize, sorghum, and soybean, respectively. Maize yield benefited from the precipitation change almost throughout the region, but the highest yield gains were found in South Dakota and North Dakota (up to 20%). Sorghum yield impacts were found mostly in Kansas, Nebraska, and Colorado with negative impacts in northwest Kansas and positive impacts elsewhere. Lastly, soybean yield impacts were primarily found in central Iowa (both positive and negative). Finally, while considering counties where both temperature and precipitation had significant impacts, we found climate-induced net yield gains of 5.4%, 1.5%, and 0.4% for maize, sorghum, and soybean, respectively.

Differences in temperature- and precipitation-induced yield impacts existed when irrigated and non-irrigated conditions were considered. Overall, non-irrigated crops exhibited greater magnitudes of yield impacts, in case of both temperature and precipitation impacts. On average, among the same counties, temperature-induced non-irrigated maize yield gains were 52% greater than temperature-induced irrigated maize yield gains, while temperature-induced non-irrigated sorghum yield losses were 98% greater than temperature-induced irrigated sorghum yield losses (no common impacted counties were found for irrigated and non-irrigated soybean). Precipitation-induced yield gains were greater for non-irrigated maize than irrigated maize by 228%, and the precipitation induced yield losses were lower for non-irrigated maize than irrigated maize by 57%. Similarly, precipitation induced yield gains for non-irrigated sorghum were greater than irrigated sorghum by 287%; however, the precipitation-induced yield losses were higher for non-irrigated than irrigated sorghum by 153%. Finally, precipitation-induced yield gains were higher for non-irrigated soybean than irrigated soybean by 372%, while there were no common counties where significant precipitation-induced gains were found for both irrigated and non-irrigated counties.

### Role of Climate-Induced Yield Trends in Overall Yield Trends

As discussed earlier, there are several other factors, in addition to climate, that play a role to determine yield trends (in the past or future). Assessing how the impacts of climate trends compare to all these other factors is important and is also one of our scientific questions. We calculated this indicator as a ratio of the climate-induced yield trend by the overall yield trend (from the county yield time series) for each county. This metric serves to emphasize the importance of climate relative to all other yield-influencing factors. The metric is interpreted as climate effect in the equivalent number of years of overall yield gains. For instance, a value of −0.05 means that 20 years of the trend in climate (temperature or precipitation) is equivalent to a setback of 1 year of technological gains, and similarly a value of +0.2 means that 5 years of the trend in climate is equivalent to advancement of 1 year of technological gains.

The measure reveals high degree of variation across the counties owing to the differences in both yield trends of the crops and climate impacts on them. The metric’s average regional magnitudes for each crop are presented in Table 1. While temperature-induced impacts accelerated yield trends in maize (it takes 47 years to advance a year of technological gains), they slowed sorghum (takes 6 years to setback a year of technological gains) and soybean (takes 91 years to setback a year of technological gains) yield trends. However, in case of precipitation-induced impacts, yield trends were accelerated for the three crops, but with varying magnitudes (51, 31, and 30 years to advance a year of technological gains for maize, sorghum, and soybean, respectively). Table 1 also presents these magnitudes for irrigated and non-irrigated conditions, but a comparison might be challenging as the yield growth rates as well as the geographic regions are subject to variations among irrigated and non-irrigated crops.

## Discussion

This study builds upon a comprehensive approach to realize the impacts of climate variability on crop yields and production by addressing a series of constituent questions in a stepwise fashion. Crop yields were studied to discover the trends and inter-annual variability during the 4.6 decades period over the Great Plains region. A key observation was high CV in crop yields for some regions such as North Dakota and South Dakota, which point towards the facts that areas with lower yields or less contribution to regional production demonstrate higher inter-annual variation than the key producing regions such as Nebraska, Kansas, and Iowa. This finding has also been reported on U.S. national and global scales, respectively^18,21^. Overall, soybean yields exhibited lowest variability relative to maize and sorghum. *Leng et al*.^18^ showed that similar spatial patterns exist in maize and soybean yield variability in the Great Plains region, although their analyses differed from ours in terms of: (a) the use of standard deviation to interpret crop yield variability, and (b) the period of analyses (1983–2012). Stability in crop yields is important, especially now than ever, when demands for food, feed, and fiber are increasing^22^ due to rapidly growing global population, and productivity rates have been lagging^23^. Crop yield variability, and hence variability in production, can affect national food stocks, spikes in food prices, and the livelihood of the citizens and other stakeholders involved. Therefore, the regions which contribute largely to national production should ideally be more resilient to variability to avoid these ramifications as even small levels of variability can affect productivity drastically. At the same time, greater variability in the regions of less consequence to regional or national production, would result in similar impacts in the local food availability scenario of these regions. Hence, our efforts to quantify crop yield variability in this study generate critical information on the state of these regions in the context of food security and quantitatively emphasizes that regions differ in their susceptibility to factors that can affect crop yields.

Further, we establish how much of the crop yield variability is explained by climate and its dynamics across the spatial domain. We found that there was considerable number of counties where climate explained more than 20% of the crop yield variability. The average magnitudes of CEYV of 18%, 23%, and 23% in qualifying counties translate to staggering fluctuations of 32 million tons, 3 million tons, and 10 million tons of regional maize, sorghum, and soybean crop production, which can have tremendous local, regional, and national economic as well as social implications. *Leng et al*.^18^ demonstrated similar spatial patterns in CEYV for the Great Plains region, when maize and soybean yield was investigated as a function of precipitation and temperature only (excluding radiation). It was discovered that contribution of counties to regional food production is an important indicator to employ while assessing climate impacts. For example, we found that precipitation alone explained sorghum yield variability in 17% of the total Great Plains counties. However, these counties are responsible for 49% (almost half) of the regional sorghum production, which is of much larger proportion than the proportion of number of counties. This is also true for soybean, where precipitation explains yield variability in 18% of the counties, which constitutes 32% (almost a third) of regional production. Thus, reporting merely proportion of affected counties, rather than proportion of crop production affected might lead to misinterpretation of actual representation of magnitude of climate-explained yield variability. We suggest that our study is novel in the light of discovering and highlighting the critical importance of this course of action and recommends using this measure in the future for more robust and realistic determinations of climate vs. crop production dynamics.

The climate-driven impacts in the region were shown to be variable across crop types and also geographically for a particular crop. Temperature-induced impacts were beneficial for maize yields, but negative for sorghum and soybean yields, with sorghum yields suffering by much higher magnitudes. Although crop yields were negatively affected by the temperature rise (sensitivity), maize was worst affected per 1 °C increase, followed by soybean and sorghum. However, the actual impacts realized for each crop, as explained earlier, is a function of yield sensitivity to a fixed change in climate and the actual observed climate trends in the crop growing regions. Both maize and soybean growing regions showed cooling (maize regions cooler than soybean regions); however, sorghum growing regions were subjected to substantial warming, which lead to negative temperature-induced impacts on sorghum yields, and positive impacts on maize yields. Similarly, all crops benefitted from a unit rise in precipitation; however the magnitudes of the sensitivity varied, with sorghum being the most sensitive, followed by maize and soybean. The observed precipitation trends were highest for maize growing region, followed by soybean and the least for sorghum, although all regions showed wetting trends. The resultant precipitation induced impacts were hence, maximum for maize, followed by sorghum and soybean. Overall, the regional effect of climate (combined temperature and precipitation) was shown to be beneficial to maize and soybean yields (although low magnitude) and slightly detrimental for sorghum. This is primarily due to the positive precipitation impacts countering (or advancing) the negative (or positive) temperature-induced impacts. Despite evaluating the climate impacts for the entire region, we still strongly suggest that the county-specific impacts be given attention while studying climate impacts as these can be, and have been shown to be substantially different than regional magnitudes. Hence, different regions have to be investigated in terms of their susceptibility/robustness against changes in climate to develop robust and meaningful, and realistic strategies to mitigate climate’s negative impacts on crops and the sensitivity and climate impacts maps can serve considerably in this regard.

Irrigated and non-irrigated yields differed in all the metrics employed in this study when investigating and demonstrating the impacts of climate variability on crop yields. First, the variability (CV) in latter was 77% (maize), 69% (sorghum), and 63% (soybean) greater than the former. Further, the CEYV in non-irrigated crops was 436% (maize), 160% (sorghum), and 728% (soybean) higher than irrigated crops, with the non-irrigated crop yield variability largely explained by precipitation alone and both precipitation and temperature, whereas temperature alone explained irrigated crop yield variability. Irrigation also decreased the sensitivity of crops to temperature rise, especially for maize, and increased sensitivity of all crops to precipitation rise. Finally, precipitation and temperature-induced actual yield impacts were more beneficial for non-irrigated maize, sorghum, and soybean, with the only exception of temperature and precipitation-induced sorghum yield losses. All these findings suggest that irrigated systems are generally robust (mitigates against negative impacts of temperature and/or precipitation change) than non-irrigated systems. These differences have also been reported on global scales^24,25^. This could be the result of: (i) irrigation reducing impacts of extreme temperatures, hence reducing or eliminating the water stress, which could otherwise reduce the yield substantially, (ii) greater transpiration rates (higher water availability) leading to evaporative cooling in the crop canopy microclimate, and (iii) greater yields due to greater magnitude of transpiration, because transpiration and grain yield are strongly and linearly correlated. Since sorghum tends were observed to be less than irrigated maize, the effects of irrigation weren’t detectable as much as for maize, which implies that effect of irrigation may be variable in certain crops due to the variations in irrigation timing and amount and irrigation method used in practice.

Any empirical model-based study has its limitations and likewise, we have identified some of these in the framework of our study. In addition to temperature and precipitation variability, there is a range of other factors that can affect crop yields such as solar radiation, wind speed, extreme temperature and precipitation, soil moisture availability (although we somewhat addressed this through irrigated vs. non-irrigated yield analyses), dry and wet spells, nutrient availability, conservation tillage, mulching and multiple cropping. Moreover, our study does not address effects of elevated carbon dioxide on crop yields. A 0.065% increase in C_3_ crop yields per ppm of CO_2_ increase and a negligible increase in C_4_ crop yields, because of the photosynthetic pathway for C_4_ crops being independent of ambient CO_2,_ has been reported^26,27^. Hence, the effect of elevated CO_2_ would be an important factor to consider in C_3_ crops such as soybean (maize and sorghum are C_4_ crops), which is outside the scope of our study. The county-specific statistical model(s) that have been employed in the study are chosen assuming linear climate-crop yield relations, which might not be true under certain conditions. As shown by studies^14,24^, crop growth has been shown to respond non-linearly to climate. Also, we did not account for changes in crop spatial distribution pattern, which has recently been shown to modulate the response of crop yields to climate and ignoring these changes could possibly result in biased estimates of climate change impacts^28^. Measures to quantify and reduce sampling uncertainty were not adopted in this study. However, accounting for sampling uncertainty is crucial because empirical models such as ours are dependent on finite observational records^28^. Nevertheless, we estimated our county-specific multiple linear regression model uncertainty using a simple root mean squared error (RMSE) for each county. Averaged over the counties where at least one of the climate factors significantly explained yield variability, the RMSE of the linear model relative to mean crop yields were 16%, 20% and 15% for maize, sorghum and soybean, respectively. We also do not address covariability of climate variables, which has also been demonstrated to have a role in estimating accurate sensitivity of crop yields to climate factors^18^. Finally, we did not consider the adaptations that have possibly already been taken (although our observations indicate to a very limited extent, at least in the Great Plains region) by the producer community against climate change such as planting date shifts, adoption of newer drought-tolerant hybrids, cultivars, varieties, regional crop pattern shifts, and other management strategies.

Our study, hence is a scientific, data-driven, and informative guide to evaluate and quantify climate’s role in yield variability of maize, sorghum, and soybean (three of the major grain commodities) grown in the U.S. Great Plains and further quantifies the actual impacts realized by this contribution in the constituent counties. This can be used to guide future research efforts in the regions that are shown to be critical in terms of climate impacts and inform concerned natural resources and policy agencies and decision makers in the region.

## Material and Methods

### Crop Yield and Climate Datasets

County-level crop yield datasets were obtained for maize, sorghum, and soybean for the period of 1968–2013 from the United States Department of Agriculture-National Agricultural Statistics Service (USDA-NASS). The beginning date of the study period (1968) was based on the start of the state level crop yield records. The earliest year with crop yield records available for all nine states studied was 1968 and, hence was chosen as the beginning of the study period. The USDA-NASS obtained yield data were reported in bushels per acre of wet weight. To make the data convenient for our use as well as for the greater scientific community, conversions were made to kilogram per hectare on a dry weight basis (adjusting to standard 15.5% grain moisture content), by using conversion factors of 62.77 for maize and 67.25 for soybean. The analysis on crop yield variability was limited to counties with data available for least 60% of the study period. It should be noted that the sorghum yield dataset was highly discontinuous prior to 1972 and following 2007 for majority of the counties, hence all analysis for sorghum was conducted for a 36-year period instead (1972–2007). Daily weather dataset consisting of maximum air temperature, minimum air temperature, and precipitation were obtained from the Global Historical Climatology Network (GHCN) provided through the National Climatic Data Centre of the National Oceanic and Atmospheric Administration (NCDC-NOAA). The GHCN datasets are subjected to rigorous quality assurance reviews. Historical daily weather datasets for the period 1968–2013 were obtained for over 800 weather stations distributed over the study area. The selection is performed so that all the weather stations selected possess regular data during the temporal span of 46 years. Out of these sites, 672 sites are geographically located in nine states studied. The remainder of the sites was selected from the surrounding states along the boundaries of the study area, specifically Arkansas, Idaho, Illinois, Louisiana, Missouri, Montana, Minnesota, New Mexico, Utah, and Wisconsin to perform accurate and continuous interpolation of various variables along the edges of the study area. Lastly, the boundary datasets for various governmental units such as states and counties that were used to aid in the analysis and representation in the GIS environment were obtained from the USDA-Geospatial Data Gateway.

### Development of County-Based Climate Data

For the purpose of exploration and investigation of impacts of climate on the agricultural crop yields, it is crucial that both datasets (climate and crop yields) are at the same spatial and temporal scales, hence rendering them suitable for inter-comparison. These scales have to be appropriate in terms of space and time so that the resulting climatic impacts are represented in a detailed, consistent, and relatable manner. In this study, a county has been chosen as an appropriate scale to investigate and report the findings. This is because several other sets of information such as commodity prices, crop yields, fertilizer inputs and management practices are reported on county-level basis. Thus, quantifying climate impacts on agricultural production on a county basis results in increased potential for the application of these findings by the state and federal agencies working in the disciplines of agriculture and natural resource conservation. Air temperature and precipitation surfaces (datasets) used in this study were developed by *Kukal and Irmak*^16^ using over 800 sites for inverse distance weighing (IDW) interpolation technique in ArcGIS 10.2. We computed zone-based (counties) values from these spatial datasets using zonal statistics tool in ArcGIS 10.2. These operations resulted in a database of county-averaged growing season mean temperature and growing season total precipitation for all 834 counties in the study region for the period of 1968–2013, which is consistent with the crop yield dataset.

### Trends in Climate and Crop Yields

We focused on the trends that occurred in crop yields and growing season climatic variables (average air temperature, T_avg_, and precipitation, P) during the period of 1968–2013. Trends were calculated for county level maize, soybean, and sorghum yields (kg ha^−1^ yr^−1^) and county averaged T_avg_ (°C yr^−1^) and P (mm yr^−1^) during the crop growing season (May 1^st^- September 30^th^) using linear regression analysis. Qualitatively, these trends in yield and climate can be realized as the direction of change in these values over the 46-year period, whereas in quantitative terms, the trends are slope of the linear regression analysis performed with yield or climate on the ordinate and the time (year) on the abscissa. This process resulted into 2,502 regressions for yield (834 counties × 3 crops) and 1,668 regressions for climate (834 counties × 2 variables). The outcomes of these regression analyses, including both directions and magnitudes of the trends were represented using maps, to better observe the trends in a spatial context, and acknowledge the extent of variability that occurs in both yield and climate change on spatial scales.

### Estimation of Climate Contribution towards Crop Yields

A series of steps and computations were involved in quantifying the contribution of climate towards trends in crop yields (climate-driven yield trends). Since the climate dataset was complete in all regards (all 834 counties and all 46 years), the analyses were restricted by the availability of crop yield records. It was decided that the analyses be performed for only those counties which had continuous crop yield data for all years during the study period (1968–2013). Since the analysis was primarily statistical, missing data were not included in the analyses keeping in mind the potential errors it can introduce into the analyses. The following procedure outlines the constituent steps involved:

- The county-level crop yields were detrended using linear regression, with crop yield as the dependent variable and time (year) as the independent variable. This was done to remove the effects of non-climatic variables from the raw time series of yield records. Primarily, the non-climatic variables include technological improvements, genetic advancements, and crop and soil management improvements. This step considers inter-annual sensitivity to climate/weather factors as a proxy for sensitivity to long-term changes in climate, which is widely accepted practice for scientific studies that have similar natures to ours^29^. Detrending results in crop yield residuals (kg ha^−1^ yr^−1^), which would be used in the further calculations.
- Similarly, the county level climate variables (growing season T_avg_ (averaged from May 1^st^ to September 30^th^) and growing season total P (summed from May 1^st^ to September 30^th^) were detrended using each of the climate variables as the dependent variables and time as the independent variable. Consequently, this resulted in T_avg_ and P residuals (°C yr^−1^ and mm yr^−1^)
- Using multiple linear regression, the yield residuals were regressed against climate residuals. The purpose of this step was the evaluation of sensitivity of yields to individual climate variables. The resulting slope coefficients from these analyses represent yield increments per unit change (or sensitivity) in the T_avg_ (kg ha^−1^ °C^−1^) and P (kg ha^−1^ mm^−1^). Also, the coefficient of determination (R^2^) for the regression provides information about what magnitudes of variability in yields can be explained by variability in climate (i.e., temperature and precipitation) for different counties (or climate explained yield variability, CEYV). Moreover, statistical significance of the impacts of individual climate variables on yields was also tested at 90% confidence interval.
- The multiple regression coefficients derived from afore-explained steps were multiplied with the climate trends (°C yr^−1^ or mm yr^−1^) obtained during the 46-year period to derive the impacts of temperature and precipitation to yield trends (kg ha^−1^ yr^−1^). Further, total change in yield during the study period that was attributed to climate was calculated by multiplying the obtained values by the number of study years (46).
- Mean county-level yields during the study period were calculated to represent average levels of yields obtained in a particular county. The contribution of individual climate variables towards yield trends were represented (in percent) as the ratio of absolute change in climate-attributed yield to the county-average yield as well as region-average yield. This was done to represent the proportion of changes in yields due to climate trends relative to average yield of a county and whether the climate impact on yield was positive or negative.
- Finally, the climate-induced yield trend was divided by overall yield trend for the period 1968–2013 for each relevant county to quantify the relative importance of climate in the overall yield trends.

All the maps that represent various indicators derived were developed using ESRI ArcMap version 10.4.1.

## References

[1] Kucharik CJ, Ramankutty N (2005). Trends and variability in US corn yields over the twentieth century. Earth Interact..

[2] Duvick DN, Cassman KG (1999). Post–green revolution trends in yield potential of temperate maize in the North-Central United States. Crop Sci..

[3] Andresen JA, Alagarswamy G, Rotz CA, Ritchie JT, LeBaron AW (2001). Weather impacts on maize, soybean, and alfalfa production in the Great Lakes region, 1895–1996. Agron. J..

[4] Duvick, D. Genetic rates of gain in hybrid maize yields during the past 40 years. Maydica (1977).

[5] Duvick, D. N. Genetic contributions to advances in yield of US maize. Maydica (Italy) (1992).

[6] Tester M, Langridge P (2010). Breeding technologies to increase crop production in a changing world. Science.

[7] Kucharik CJ (2008). Contribution of planting date trends to increased maize yields in the central United States. Agron. J..

[8] Stocker, T. F. *et al*. IPCC, 2013: climate change 2013: the physical science basis. Contribution of working group I to the fifth assessment report of the intergovernmental panel on climate change (2013).

[9] Nicholls N (1997). Increased Australian wheat yield due to recent climate trends. Nature.

[10] Peng S (2004). Rice yields decline with higher night temperature from global warming. Proc. Natl. Acad. Sci. USA.

[11] Tao F, Yokozawa M, Xu Y, Hayashi Y, Zhang Z (2006). Climate changes and trends in phenology and yields of field crops in China, 1981–2000. Agric. For. Meteorol..

[12] Tao F, Yokozawa M, Liu J, Zhang Z (2008). Climate–crop yield relationships at provincial scales in China and the impacts of recent climate trends. Climate Research.

[13] Lobell DB, Schlenker W, Costa-Roberts J (2011). Climate trends and global crop production since 1980. Science.

[14] Lobell DB, Bänziger M, Magorokosho C, Vivek B (2011). Nonlinear heat effects on African maize as evidenced by historical yield trials. Nature Climate Change.

[15] Lobell DB, Asner GP (2003). Climate and management contributions to recent trends in US agricultural yields. Science.

[16] Kukal M, Irmak S (2016). Long-term patterns of air temperatures, daily temperature range, precipitation, grass-reference evapotranspiration and aridity index in the USA Great Plains: Part I. Spatial trends. Journal of Hydrology.

[17] Kukal M, Irmak S (2016). Long-term patterns of air temperatures, daily temperature range, precipitation, grass-reference evapotranspiration and aridity index in the USA Great Plains: Part II. Temporal trends. Journal of Hydrology.

[18] Leng G, Zhang X, Huang M, Asrar GR, Leung LR (2016). The Role of Climate Covariability on Crop Yields in the Conterminous United States. Sci. Rep..

[19] Leng G (2017). Recent changes in county-level corn yield variability in the United States from observations and crop models. Sci. Total Environ..

[20] Leng G (2017). Evidence for a weakening strength of temperature-corn yield relation in the United States during 1980–2010. Sci. Total Environ..

[21] Ray DK, Gerber JS, MacDonald GK, West PC (2015). Climate variation explains a third of global crop yield variability. Nat. Commun..

[22] Foley JA (2011). Solutions for a cultivated planet. Nature.

[23] Ray DK, Mueller ND, West PC, Foley JA (2013). Yield trends are insufficient to double global crop production by 2050. PloS one.

[24] Schlenker W, Roberts MJ (2009). Nonlinear temperature effects indicate severe damages to U.S. crop yields under climate change. Proc. Natl. Acad. Sci. USA.

[25] Lobell DB, Gourdji SM (2012). The influence of climate change on global crop productivity. Plant Physiol..

[26] Ainsworth EA, Leakey AD, Ort DR, Long SP (2008). FACE-ing the facts: inconsistencies and interdependence among field, chamber and modeling studies of elevated [CO_2_] impacts on crop yield and food supply. New Phytol..

[27] Leakey AD (2009). Rising atmospheric carbon dioxide concentration and the future of C_4_ crops for food and fuel. Proc. Biol. Sci..

[28] Leng G, Huang M (2017). Crop yield response to climate change varies with crop spatial distribution pattern. Scientific Reports.

[29] Lobell DB, Field CB (2007). Global scale climate–crop yield relationships and the impacts of recent warming. Environmental research letters.

